# Complete Genome Sequencing of the Attenuated Strain Mycoplasma synoviae 5-9

**DOI:** 10.1128/MRA.00981-21

**Published:** 2021-12-09

**Authors:** Xiaorong Zhang, Yang Chen, Zehua Wei, Shuang Ma, Mengjiao Guo, Dianfeng Chu, Chengcheng Zhang, Yongzhong Cao, Yantao Wu

**Affiliations:** a Jiangsu Co-Innovation Center for the Prevention and Control of Animal Infectious Disease and Zoonoses, College of Veterinary Medicine, Yangzhou University, Yangzhou, Jiangsu, China; b State Key Laboratory of Genetically Engineered Veterinary Vaccines, Qingdao Yebio Biological Engineering Co., Ltd., Qingdao, Shandong, China; c The International Joint Research Laboratory of Agricultural and Agri-product Safety, Yangzhou University, Yangzhou, Jiangsu, China; University of Maryland School of Medicine

## Abstract

Here, we present the complete genome sequence of Mycoplasma synoviae strain 5-9. Strain 5-9 was attenuated by chemical mutagenesis from a field strain isolated from egg breeders in Ningxia, China. It was completely sequenced and its genome annotated; it is presented with the relevant data as a potential vaccine candidate.

## ANNOUNCEMENT

Mycoplasma synoviae is an important pathogen infecting chickens, causing acute or chronic respiratory diseases, infectious synovitis, air sacculitis (1–3), and eggshell apex abnormalities (4). Ningxia/2017-1 is the virulent parent strain of 5-9, isolated in Ningxia, China. M. synoviae isolation and identification using *Mycoplasma* broth (MB) and *Mycoplasma* agar (MA) were conducted as previously described (5). A Ningxia/2017-1 culture was exposed to 100 μg/ml N-methyl-N′-nitro-N-nitrosoguanidine (NTG) for 15 min and continuously passaged 5 times in 10 μg/ml NTG at 33°C before being plated onto MA. Single colonies were selected to grow in MB at 33°C (6).

High-quality genomic DNA (optical density at 260/280 nm [OD_260/280_] = 1.8 ∼ 2.0) from 5-9 was extracted for Illumina and ONT sequencing using a bacterial genomic DNA kit (CWBIO Biotech, China) until the color changed from red to orange-yellow when cultured at 33°C and passaged 2 times in MB (pH 6.9). For next-generation sequencing (NGS), paired-end libraries with insert sizes of ∼400 bp were prepared using the Illumina TruSeq Nano DNA high-throughput library prep kit. The purified DNA was sheared into smaller fragments of the desired size (Covaris). Finally, the qualified Illumina paired-end library was used for Illumina NovaSeq 6000 sequencing (2 × 150-bp format; Shanghai BIOZERON Co., Ltd., China). A total of 2,333 Mb raw data was produced, and we obtained 2,222 Mb clean data after filtering out the low-quality reads using Trimmomatic (7) (http://www.usadellab.org/cms/?page=trimmomatic) (Table 1). The raw paired-end reads were trimmed and quality controlled using Trimmomatic v0.36 (7) (http://www.usadellab.org/cms/?page=trimmomatic) (parameters, SLIDINGWINDOW:4:15 MINLEN:75).

**TABLE 1:** Genome assembly, Nanopore sequencing, and Illumina sequencing data

Sample	Total length (bp)	*N*_50_ length (bp)	G+C content (%)	Coverage (×)	Nanopore sequencing			Illumina sequencing			
								Raw data		Clean data	
					No. of reads	No. of bases	Avg length (bp)	Total no. of reads	Total size (Mb)	Total no. of reads	Total size (Mb)
5-9	786,872	786,872	28.47	1,015	125,369	798,728,344	6,371	15,553,532	2,333	14,881,648	2,222.2

A Nanopore library was prepared using the ONT 1D ligation sequencing kit (SQK-LSK108) with the native barcoding expansion kit (EXP-NBD103). At least 1 μg DNA was treated with the end-repair/dA-tailing module, but the DNA was eluted in 24 μl following AMPure XP bead cleanup. Following the barcode ligation reaction, the DNA was cleaned again using AMPure XP beads and elution in 10 μl. The run was performed on a MinION MK1b device using the NC_48 h_Sequencing_Run_FLOMIN106_SQK-LSK108 protocol.

The Illumina data were used to evaluate the complexity of the genome and correct the Nanopore long reads. First, we used ABySS v2.02 (8, 9) (http://www.bcgsc.ca/platform/bioinfo/software/abyss) to perform the genome assembly with multiple kmer parameters and obtain optimal assembly results. Second, Canu v2.0 (10) (https://github.com/marbl/canu) was used to assemble the Nanopore corrected long reads. Finally, GapCloser v1.12 (11) (https://sourceforge.net/projects/soapdenovo2/files/GapCloser/) was applied to fill the remaining local inner gaps and correct single-base polymorphisms for the final assembly results. The complete circularized genome was drawn using Circos v0.64 (12) (http://circos.ca/). Default parameters were used for all software unless otherwise specified. The consensus assembly generated one contig of 786,872 bp (1,015-fold coverage). The G+C content was 28.47% (Table 1).

Gene models were identified using GeneMark. Then, all gene models were searched using blastp against the NCBI nonredundant (NR), Swiss-Prot (http://uniprot.org), KEGG (http://www.genome.jp/kegg/), and COG (http://www.ncbi.nlm.nih.gov/COG) databases. Additionally, tRNAs were identified using tRNAscan-SE v1.23 (13) (http://lowelab.ucsc.edu/tRNAscan-SE), and rRNAs were determined using RNAmmer v1.2 (14) (https://services.healthtech.dtu.dk/service.php?RNAmmer-1.2). There were 652 protein-coding genes and 44 RNA genes (34 tRNAs, 3 5S rRNAs, 2 16S rRNAs, 2 23S rRNAs, and 3 noncoding RNA [ncRNA] genes).

### Data availability.

The complete genome sequence and annotation of M. synoviae strain 5-9 have been deposited at GenBank under accession number CP083748. The raw data were deposited in the Sequence Read Archive (SRA) database under the accession numbers SRR16005423 (Oxford Nanopore) and SRR16005422 (Illumina). The BioProject accession number is PRJNA763075. The BioSample accession number is SAMN21422653.
